# Polyglycolic acid sutures embedded in abdominal acupoints for treatment of simple obesity in adults: a randomized control trial

**DOI:** 10.1186/s13020-019-0258-5

**Published:** 2019-09-18

**Authors:** Li-Shu Chen, Yue-Ying Li, Hao Chen, Bo-Wen Liu, Da-Wei Wang, Yong-Hua Zhao

**Affiliations:** 1 Ningbo College of Health Sciences, Ningbo, 315100 China; 2 Tianjin Medical University Cancer Institute and Hospital, National Clinical Research Center, Key Laboratory of Cancer Prevention and Therapy, Tianjin’s Clinical Research Center for Cancer, Tianjin, 300060 China; 3 Nanjing University of Chinese Medicine, Nanjing, 210046 China; 4 Department of Neurology, Nanjing Drum Tower Hospital Clinical College of Traditional Chinese and Western Medicine, Nanjing University of Chinese Medicine, Nanjing, 210008 China; 5 Shunde Hospital of Guangzhou University of Chinese Medicine, Shunde, 528333 China; 6 State Key Laboratory of Quality Research in Chinese Medicine, Institute of Chinese Medical Sciences, University of Macau, Taipa, Macao, 999078 Macao SAR China

**Keywords:** Polyglycolic acid sutures embedding therapy, Simple obesity, Therapeutic effects, Regulation of inflammatory response

## Abstract

**Background:**

Acupoint catgut embedding therapy characterized by acupoint, needle and catgut are superior to traditional acupuncture, due to exerting more comprehensive therapeutic efficacy. However, it is still deficient in clinical evidence for polyglycolic acid sutures, a novel biodegradable material instead of catgut, embedded for the treatment of simple obesity. In our study, we investigate the efficacy and related mechanism of polyglycolic acid sutures embedded in abdominal acupoints on simple obese persons by a randomized control trial.

**Methods:**

A total of 51 eligible participators were randomly allocated to a polyglycolic acid sutures embedding therapy (PASET) group (n = 28) or control group (n = 23). Participators in PASET group received polyglycolic acid sutures alternatively embedded in abdominal I group and II group acupoints in odd and even number therapeutic courses, and participators in control group were required to perform lifestyle modification. The duration of the study was 10 weeks.

**Results:**

It suggested that PASET significantly reduced weight, body mass index, hip circumference, waist circumference, waist/hip ratio, waist-to-height ratio and thickness of abdominal subcutaneous fat tissue compared with those before treatment (p < 0.01), but lifestyle modification only illustrated downward trend of weight (p < 0.05). Moreover, PASET group also improved the evaluated scores in aspects of physical function, self-esteem, public distress and sexual life, as well as decreased blood pressure, glycemia, low density lipoprotein, uric acid and the levels of tumor necrosis factor-alpha, interleukin-1β, and increased high density lipoprotein in comparison with those before treatment (p < 0.05), whose efficacies are superior to control group. Additionally, our results also indicate PASET is relative safe and its pain and discomfort can be tolerable.

**Conclusions:**

PASET distinctly ameliorates anthropometric data and quality of life in obese population, which associates with improvements of metabolic profile and inflammatory response. Based on the advantageous actions, we think PASET is an effective therapeutic approach to simple obesity treatment.

*Trial registration* ChiCTR, ChiCTR1800015591. Registered 10 April 2018, http://www.chictr.org.cn/showproj.aspx?proj=23258

## Background

Obesity elicited by the interaction of genetic and environmental factors is a culprit of many metabolic dysfunctions including insulin resistance, dyslipidemia, hypertension and even certain cancers [1]. Simple obesity refers to the type of obesity which associates with unhealthy lifestyles while having no other complications, e.g. diabetes mellitus or polycystic ovary syndrome [2, 3], and it is characterized by expansion of fat mass and adipocyte size, as well as lesser extent adipocyte proliferation. An epidemiological survey on 19.2 million adult participants (9.9 million men and 9.3 million women) in 186 countries suggests that the prevalence of obesity markedly ascends from 3.2% in 1975 to 10.8% in 2014 in men and from 6.4% in 1975 to 14.9% in 2014 in women. If the trends continue, by 2025 global obese ratio will reach 18% in men and surpass 21% in women [4]. According to the World Health Organization (WHO) report in 2010, at least 3.4 million adults died from overweight or obesity-related diseases each year [5]. Therefore, adiposity has become a predominant public health concern.

Although pathological mechanisms of simple obesity are complex, present consensus are metabolic regulation, neurohormonal and haemodynamic dysfunctions resulted in inflammation, lipotoxicity, apoptosis, oxidative stress, autophagy alterations and disorders of energy homeostasis and cellular metabolism [6]. Excessive adipose tissues infiltrated by various immune cells play a critical role in the production of chronic low-grade inflammation. It contributes to the onset of type 2 diabetes mellitus (T2DM) and cardiovascular complications [7]. Due to harmful features of simple obesity for health, many intervention strategies emerge as weight loss requirement. The most conventional way of weight control is lifestyle modification, which includes diet, exercise, and behavior therapy [8]. Obese populations are required to habituate regular physical activity and exercise, restrict high carbohydrate and fat intakes, and self-monitor food intake, physical activity and body weight. Although some people fail to maintain the lifestyle modification followed by recovering previous weight, lifestyle-treated persons still display low incidence of T2DM, showing beneficial effect on long-term health [9]. Additionally, studies also indicate the change of diet and/or exercise, as well as cognitive behavioral therapy for obese individuals do not produce distinct or sustainable weight loss. It is necessary to develop anti-obesity drugs by decreasing the consumption or absorption of food, and/or increasing energy expenditure [10]. However, due to unacceptable side effects, some new anti-obesity drugs have to be withdrawn. Traditional pharmacological monotherapies for obesity usually evoke counter-regulation, so new anti-obesity drugs not only possess sustained efficacy of weight loss, but also improve comorbidities by targeting multi-biological mechanisms in future. As an invasive approach, bariatric surgery quickly and effectively decreases adipose tissue for weight loss, but it is easy to elevate perioperative mortality and surgical complications. Thereby, operative treatment is regarded as only suitable for the treatment of morbidly obese [11]. Until now, researchers are still looked for satisfied therapy for anti-obesity.

From the view of Chinese medicine theory, the etiology of obesity mainly associates with improper lifestyle resulted in the production of Phlegm-damp due to spleen Qi deficiency. Accumulated Phlegm-damp subsequently transforms to stagnation heat, phlegm heat, dampness heat and stasis heat, which leads to the status of chronic inflammation in simple obesity [12]. Chinese medicine has the characteristic of holism, so systemic effects of Chinese materia medica or acupuncture contribute to regulation of obesity body. However, due to unclear compositions and vague mechanisms for Chinese materia medica, it hinders their wide application. For example, aristolochic acids and similar compounds from *Aristolochia* and related plants are mutagenesis attribution to liver cancers in Asia population [13]. While acupuncture is relatively safe therapeutic method and has been widely used in clinical practice [14]. Numerous clinical data have demonstrated that acupuncture for simple obesity is an effective method by regulation of endocrine system, promotion of digestion, attenuation of oxidative stress and modulation of relevant metabolic molecules [6, 15]. Acupoint catgut embedding (ACE) is a special acupuncture technology, whose characteristics including acupoint, needle and catgut are available to exert more comprehensive therapeutic efficacy compared with acupuncture alone. A meta-analysis of 43 trials within 3520 patients shows that ACE effectively reduces body weight, hip circumference (HC) and waist circumference (WC), and the efficacies are greater than/or equal to other kinds of acupuncture and drugs and nondrug therapy. Even in some sense, ACE may be more advantageous to obese treatment [16]. However, it is still deficient in high-quality clinical research evidence and evaluation of ACE safety for treatment of simple obesity [17]. In present study, polyglycolic acid sutures, as a novel biodegradable material, are used to embed in abdominal acupoints of obese populations instead of common catgut. Subsequently, we assess the efficacy and disclose corresponding mechanisms of the polyglycolic acid sutures embedding therapy (PASET) on simple obesity.

## Materials and methods

### General data and study design

**Fig. 1:**
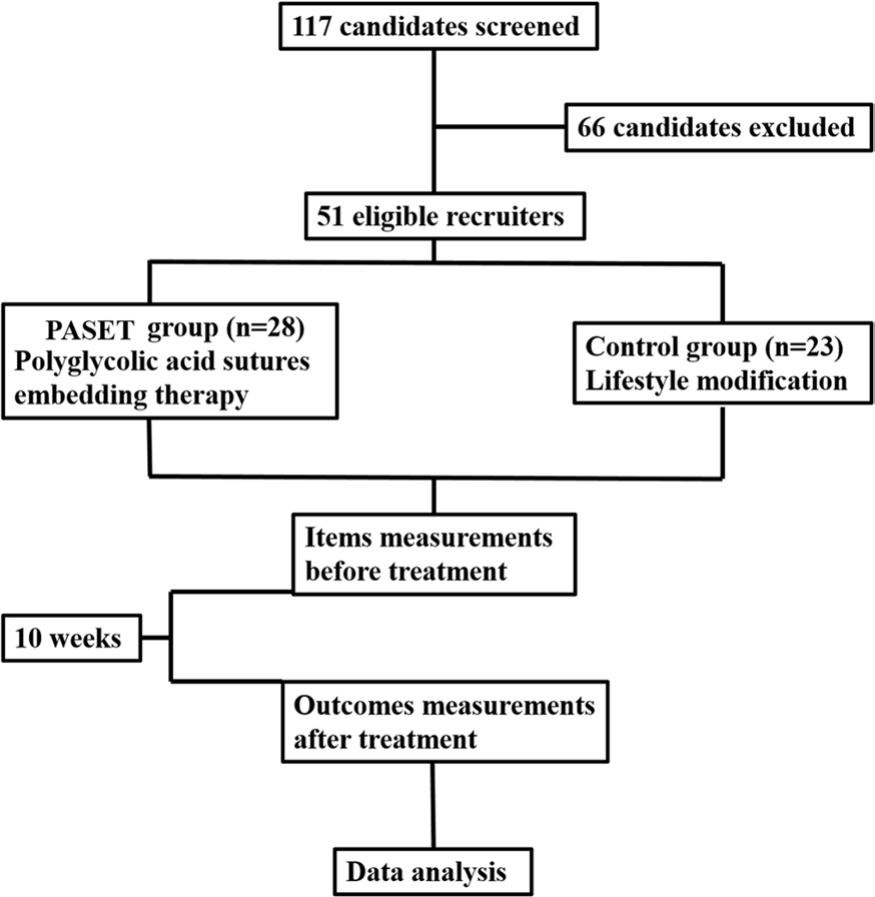
The flow diagram of the study procedure

All of the candidates were recruited from the department of anti-obesity at Shunde Hospital of Guangzhou University of Chinese Medicine (Guangdong, China) from May, 2018 to May, 2019. This clinical trial was approved by ethical committees of Shunde Hospital of Guangzhou University of Chinese Medicine and the Second Affiliated hospital of Nanjing University of Chinese Medicine (Registration number: ChiCTR1800015591). The flow diagram of the study procedure is shown in Fig. 1.

### Diagnostic criteria

The standardized protocol of anthropometric measurements in obesity was administrated according to WHO definition. Candidates needed to conform to body mass index (BMI) ≥ 28 kg/m^2^, waist/hip ratio (WHR) > 0.9 in men or WHR > 0.85 in women, and waist-to-height ratio (WtHR) > 0.5.

### Inclusion criteria and group

Candidates ages ranged from 25 to 42 years, and they conformed to the obesity diagnostic criteria and written informed consent on a voluntary basis, as well as had not significant obese comorbidities. Finally, there were 51 obese persons were recruited and randomly allocated to two groups by the random numbers: PASET group, 28 cases; and control group, 23 cases. Before treatment, the age, height and BMI of the two groups were compared (Table 1), and there was no statistical difference (p > 0.05).

**Table 1:** Characteristics of the participants

Group	Age (years)	Height (cm)	BMI (kg/m^2^)
PASET (n = 28)	34.43 ± 7.63	164 ± 0.07	31.50 ± 3.56
Control (n = 23)	34.60 ± 5.21	163 ± 0.06	30.57 ± 2.48

### Exclusion criteria

1. Candidates with serious primary diseases in cardio- or cerebral-vascular disease, failure of liver and/or kidney function, infectious disease, T2DM, polycystic ovary syndrome and mental disorders;
2. Pregnancy or having pregnant wish, breastfeeding period and secondary obesity;
3. Candidates were unable to accept embedding treatment.

### Group and treatment

#### 1. Control group

Participators were required to modify their lifestyle and perform the protocols of diet control and physical exercise.

##### Hypocaloric diets

Diet plans were individualized and energy-matched according to participators’ basal metabolic rate [18]. The low-calorie diet allowed 1000–1200 kcal/day, including 50–60% carbohydrates, 20% protein, < 30% total fat, and 18 g of fiber/1000 kcal [19]. The diet protocol was maintained to ensure planned isocaloric control between diets in the whole process of study.

##### Exercise intervention

The exercise intensity was defined as a percentage of maximum heart rate (220 minus age) and an individual’s aerobic exercise intensity should be between 65 and 85% of maximum heart rate, which was regarded as the target heart rate (THR). Based on the range of participators’ age in the study, THR between 110 beats per minute (bpm) and 155 bpm are determined as the symbol of middle exercise intensity. The exercise training program was continued for 10 weeks, seven times per week and lasting 30–60 min every time.

#### 2. PASET group

The abdominal acupoints (I) zhongwan (RN12), shuifen (RN9), qihai (RN6), yinjiao (RN7), shiguan (RN5), mangshu (KI16), siman (KI14), taiyi (ST23), tianshu (ST25), daju (ST27), fujie (SP14), daheng (SP15), daimai (GB26); (II) jianli (RN11), xiawan (RN10), guanyuan (RN4), shimen (KI18), shangqu (KI17), zhongzhu (KI15), qixue (KI13), huaroumen (ST24), wailing (ST26), shuidao (ST28), daheng (SP15), fuai (SP160), daimai (GB26) were alternative selected for embedding therapy using polyglycolic acid suture. In odd number therapeutic course, polyglycolic acid sutures were embedded in I group acupoints, in contrast, II group acupoints were chose in even number therapeutic course.

The operated apparatus were disposable. 3.0 polyglycolic acid sutures were cut into 1.5–2.0 cm line segments, and the suture segments were threaded into no. 7 needle. Subsequently, the operator sterilized the abdomen with iodophor, and the needle was rapidly inserted into the selected acupoints with a 45° angled and pushed forward. Until arriving at adipose tissues layer, the needle tubing was withdrawn and the suture segments were left. Finally, the pinhole was pressed with sterilized cotton ball for a while to prevent hemorrhage, and medical gauze was applied to cover in the pinholes. The participators were told that the pinholes should keep away water. Additionally, PASET also avoided participators’ menstrual period. The treatment cycle was repeated every 10 days, and the therapeutic time continued for 10 weeks.

### Measurements of anthropometric data and blood pressure

At the beginning and end of the trial, the outcomes of weight, BMI, WC, HC, WHR and WtHR were assessed in each group. The primary outcomes of this study are weight and BMI, while the secondary outcomes are WC, HC, WHR and WtHR. Diastolic and systolic pressure were measured using automated electronic sphygmomanometer (OMRON, HEM-7211, Japan) before and after treatments.

### Evaluations of life quality

The Impact of Weight on Quality of Life Questionnaire (IWQOL-Lite) was applied to assess the changes of life quality [20]. Before and after treatment, the participators were required to fill 31 items in the questionnaire regarding the impact of obesity on physical function, self-esteem, sexual life, public distress and work. They were asked to rank items related to obesity in 5 areas, ranging from 5 “always true” to 1 “never true”, by an independent investigators who gave a detailed explanation regarding every item of this questionnaire to participators before the investigation.

### Blood glucose, uric acid, lipid profile and inflammatory cytokines

Fasting venous blood of 10 mL from the left cubital vein was taken for the measurements of glucose, uric acid, triglyceride (TG), total cholesterol (TC), high density lipoprotein cholesterol (HDL-C) and low density lipoprotein cholesterol (LDL-C), as well as tumor necrosis factor-alpha (TNF-α), interleukin-6 (IL-6), interleukin-1beta (IL-1β), interleukin-18 (IL-18), monocyte chemoattractant protein-1 (MCP-1) before and after treatment. The blood samples were centrifuged (1500*g* for 10 min) and the serum were stored at − 20 °C. Glucose, uric acid, TG, TC, HDL-C and LDL-C were detected by routine enzymatic methods, and the inflammatory cytokines were measured using enzyme-linked immunosorbent assay (ELISA). The assay kits were purchased from Elixir Canada Medicine Co. Ltd. (Hermes Criterion Biotechnology, Vancouver, Canada) and the experimental procedures were performed according to the instructions.

### Scanning the thickness of abdominal subcutaneous fat tissue by B-mode ultrasound

The thickness of abdominal subcutaneous fat tissue in two groups (n = 10) was assessed by B-mode ultrasound (ACUSON NX3 Ultrasound System, Siemens, Germany). A 15 MHz linear transducer was respectively placed perpendicular to the skin surface on the upper and lower abdomen along the midline (linea alba), where is 3 cm distant from the navel of participators with lying in a comfortable position. The fat thickness (mm) was determined as the perpendicular distance between the skin surface and the upper border of the adipose/muscle interface [21]. The operator recorded the values of abdominal two zones before and after the treatment.

### Evaluation of safety for the performance of PASET

During the process of PASET, we carefully observed the change of skin surface and evaluated patients’ pain extent using Visual Analogue Scale (VAS) [22]. In every therapeutic course, we checked if there were erythematous swelling and/or nodules to appear on the embedding sites of abdomen, as well as understood the pain feeling of participants.

### Statistical analysis

All data were analyzed using SPSS 16.0 (SPSS Inc., Chicago, IL, USA). The Kolmogorov–Smirnov test was used to examine whether variables were normally or non-normally distributed. Student t and Mann–Whitney tests were performed to compare parametric and nonparametric data between two samples, respectively. The paired t-test and Wilcoxon signed ranks tests were respectively applied to compare initial and final data in each group. Statistical significance (two-tailed) was set at *p* < 0.05 for all analyses.

## Results

### PASET improves anthropometric measurements

As shown in Table 2, in PASET group, the values of weight, BMI, WC, HC, WHR and WtHR showed significantly downward trend post-treatment compared with pre-treatment (p < 0.01), and the values of weight either in PASET group or control group before treatment distinctly descended in comparison with after treatment, suggesting PASET and lifestyle modification were able to lose weight. However, PASET more significantly ameliorated anthropometric measurement values than lifestyle modification.

**Table 2:** Comparison of anthropometric measurement values in two groups

	PASET group			Control group		
	Before treatment	After treatment	p value	Before treatment	After treatment	p value
Weight (kg)	83.05 (75.30, 92.80)	78.90** (72.73, 89.40)	0.000	83.05 (72.37, 93.15)	81.55* (71.96, 93.30)	0.021
BMI (kg/m^2^)	31.45 (28.79, 32.88)	29.88** (27.48, 30.65)	0.003	30.68 (28.68, 32.12)	30.07 (28.42, 34.64)	0.218
WC (cm)	103.10 (98.00, 107.90)	95.50** (92.95, 101.00)	0.000	101.00 (97.10, 107.25)	102.50 (96.00, 105.75)	0.775
HC (cm)	107.00 (101.03, 112.00)	101.00** (98.00, 108.50)	0.000	109.00 (104.80, 111.00)	108.60 (104.75, 114.60)	0.635
WtHR	0.59 (0.58, 0.64)	0.56** (0.53, 0.59)	0.000	0.58 (0.55, 0.59)	0.58 (0.56, 0.61)	0.081
WHR	0.96 (0.92, 1.00)	0.94** (0.93, 0.96)	0.004	0.91 (0.89, 0.98)	0.91 (0.90, 0.97)	0.653

Statistical significant difference between before and after treatment in each group (* p < 0.05, ** p < 0.01)

### PASET enhances life quality of obesity

To study whether PASET also improve life quality, we employed IWQOL-Lite questionnaire to measure participators’ life quality including five sections of physical function, self-esteem, public distress, sexual life and work. In Table 3, either total scores or every item score in PASET group after treatment were higher than those before treatment (p < 0.05), illustrating PASET notably elevated life quality in obese persons. It embodied comprehensive amelioration of physical and psychological functions. However, in control group, the scores of self-esteem and public distress after treatment were lower than those before treatment, although there was no statistical difference. It suggested lifestyle modification failed to present satisfied improvement of psychology in obesity treatment.

**Table 3:** The evaluation of life quality using IWQOL-Lite questionnaire

	PASET group			Control group		
	Before treatment	After treatment	p value	Before treatment	After treatment	p value
IWQOL-Lite total	88.00 (79.50, 103.00)	98.00** (95.00, 112.50)	0.002	85.00 (71.00, 100.00)	85.00 (77.00, 101.50)	0.616
Physical function	41.00 (34.50, 51.00)	49.00** (40.00, 51.00)	0.002	45.00 (41.00, 48.00)	49.00 (39.00, 51.00)	0.323
Self-esteem	22.00 (11.00, 29.00)	29.00** (23.50, 33.00)	0.000	22.00 (8.00, 30.00)	16.00 (7.50, 32.00)	0.874
Sexual life	18.00 (13.50, 20.00)	19.00* (16.50, 20.00)	0.048	19.00 (14.00, 20.00)	20.00 (15.50, 20.00)	0.068
Public distress	21.00 (15.50, 25.00)	21.00* (17.50, 25.00)	0.015	21.00 (13.50, 25.00)	16.00 (15.00, 23.00)	0.722
Work	19.00 (11.50, 20.00)	15.00 (14.00, 18.00)	0.302	14.00 (11.00, 20.00)	14.00 (12.50, 19.00)	0.379

Statistical significant difference between before and after treatment in each group (* p < 0.05, ** p < 0.01)

### PASET reduces glycemia, blood pressure and regulates dyslipidemias

To understand the potential efficacy of PASET on metabolic dysfunction of obesity, we detected the participators’ blood pressure and the concentrations of glucose and lipid profile. As shown in Table 4, participators’ diastolic pressure and systolic pressure showed distinctly downward after treatment compared with before treatment in PASET group (p < 0.01). The concentrations of HDL-C/LDL-C and uric acid were also significantly ameliorated after PASET, but blood glucose, triglyceride and total cholesterol did not suggest extraordinary changes. In control group, there was no notably difference about the metabolic items between before and after treatments.

**Table 4:** Glycemia, blood pressure and lipid profile before and after treatment

	PASET group			Control group		
	Before treatment	After treatment	p value	Before treatment	After treatment	p value
Diastolic pressure (mmHg)	78.00 (74.00, 95.75)	77.00** (70.00, 87.00)	0.004	75.50 (68.75, 77.75)	75.00 (71.25, 80.50)	0.294
Systolic pressure (mmHg)	117.50 (109.75, 141.50)	112.50** (108.00, 132.50)	0.005	107.50 (103.75, 120.00)	106.50 (104.25, 121.25)	0.803
Glucose (mmol/L)	5.33 (4.09, 6.43)	5.23 (4.95, 5.85)	0.796	5.25 (4.93, 5.66)	5.21 (4.96, 5.68)	0.270
Triglyceride (mmol/L)	2.48 (1.31, 4.40)	2.24 (1.01, 4.18)	0.535	1.78 (1.12, 2.60)	1.74 (1.17, 2.45)	0.208
Total cholesterol (mmol/L)	5.19 (5.12, 5.97)	5.25 (4.94, 6.22)	0.070	4.75 (4.36, 5.50)	5.03 (4.50, 5.80)	0.683
High-density lipoprotein (mmol/L)	1.12 (1.08, 1.32)	1.35** (1.00, 1.39)	0.008	1.27 (1.02, 1.50)	1.31 (1.03, 1.50)	0.972
Low-density lipoprotein (mmol/L)	3.38 (2.83, 4.15)	2.84* (2.50, 3.91)	0.049	3.02 (2.61, 3.67)	3.26 (3.03, 3.70)	0.172
Uric acid (μmol/L)	409.00 (317.00, 495.75)	326.00* (291.75, 517.75)	0.038	430.00 (341.75, 531.00)	393.00 (313.50, 474.00)	0.096

Statistical significant difference between before and after treatment in each group (* p < 0.05, ** p < 0.01)

### PASET modulates inflammatory cytokines in peripheral blood

In 1993, Hotamisligil found that TNF-α played an important role in regulation of insulin resistance in obese rats [23]. Accumulated evidences indicate that some inflammatory cytokines have been regarded as primary marker of a persistent, low-grade, inflammatory response in obesity and its associated diseases [24]. In Table 5, it showed that PASET obviously decreased the concentrations of TNF-α and IL-1β in peripheral blood after treatment compared with before treatment (p < 0.05). Unexpectedly, the concentration of IL-18 suggested slightly higher after treatment than that before treatment, although there was no statistical difference. In contrast, IL-18 level in control group reversed the changed trend. The concentrations of IL-6 and MCP-1 in PASET group after treatment showed downward trend, but the values did not present statistical significance compared with those before treatment. In lifestyle modification group, other inflammatory cytokines levels displayed mildly elevated trend except IL-18, suggesting the therapeutic strategy failed to improve low-grade inflammatory status in obese persons.

**Table 5:** The levels of inflammatory cytokines before and after treatment

	PASET group			Control group		
	Before treatment	After treatment	p value	Before treatment	After treatment	p value
TNF-α	162.76 (81.42, 201.17)	141.21* (81.31, 177.83)	0.049	157.46 (125.20, 162.94)	165.74 (133.96, 196.16)	0.116
IL-1β	174.45 (159.24, 304.91)	158.29* (132.72, 264.89)	0.023	175.96 (151.64, 211.33)	175.39 (155.83, 220.14)	0.683
IL-18	168.41 (127.39, 224.13)	179.73 (163.96, 237.69)	0.173	173.54 (107.40, 185.49)	141.97 (114.68, 190.74)	0.173
IL-6	181.41 (156.72, 272.76)	143.44 (95.14, 257.74)	0.134	142.52 (92.31, 192.52)	177.40 (92.87, 263.68)	0.158
MCP-1	220.44 (184.65, 320.10)	192.85 (180.33, 286.42)	0.796	194.04 (185.92, 233.25)	236.83 (178.53, 252.78)	0.084

Statistical significant difference between before and after treatment in each group (* p < 0.05)

### PASET decreases the thickness of abdominal subcutaneous fat tissue

**Fig. 2:**
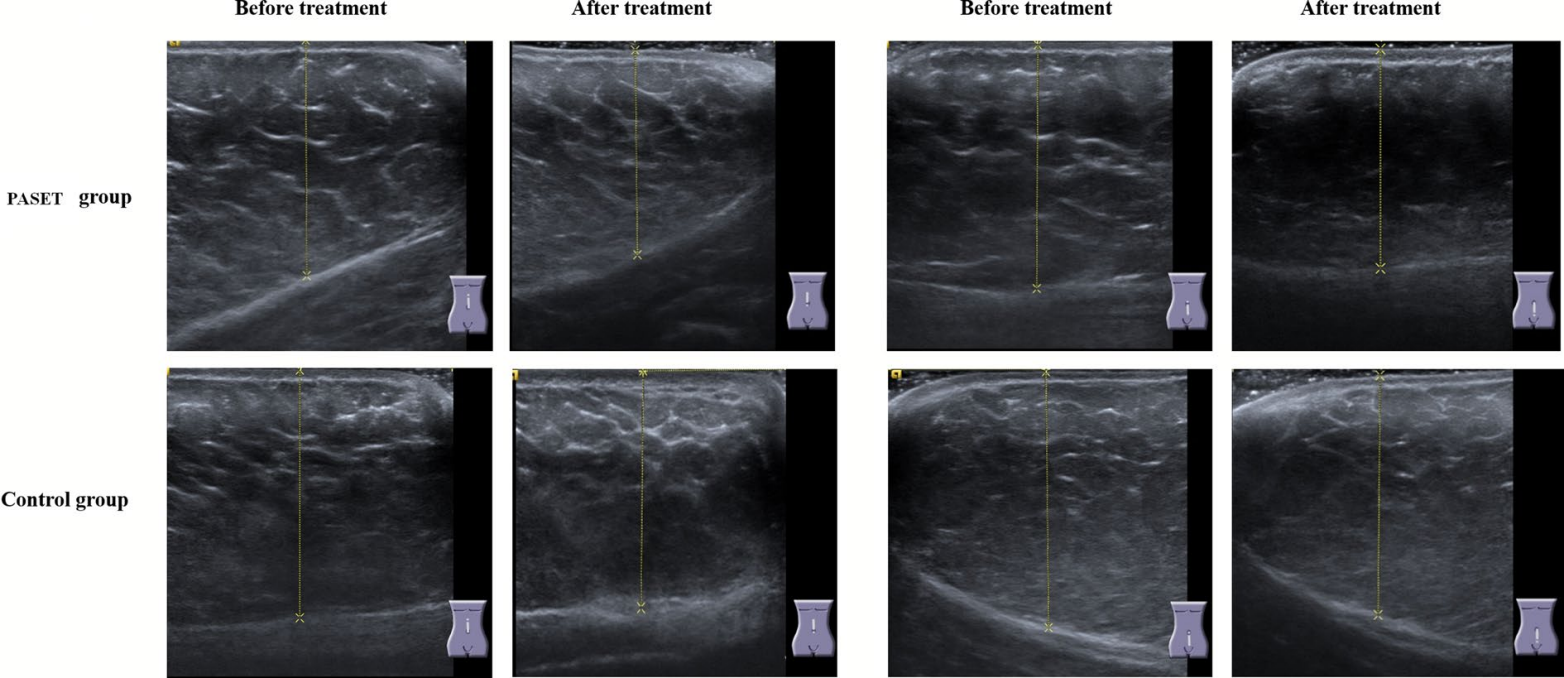
The measurement of abdominal subcutaneous fat tissue’s thickness by using B-model ultrasound. A 15-MHz linear transducer was placed perpendicular to the skin surface. Subcutaneous fat thickness (mm) was measured as the perpendicular distance from the skin surface to the upper border of the adipose/muscle interface

The abdominal subcutaneous fat tissue’s thickness was measured by using B-model ultrasound and the representative images were showed in Fig. 2. Quantitative analysis in Table 6 indicated that the thickness of upper abdominal subcutaneous fat tissue after PASET treatment was thinner than that before treatment (37.63 ± 5.82 mm vs 40.95 ± 7.03 mm, p < 0.01), and lower abdominal subcutaneous fat tissue’s thickness in treatment group had similar trend (35.37 ± 7.11 mm vs 38.86 ± 6.96 mm, p < 0.01). In contrast, the thickness in control group got slightly thicker after treatment than that before treatment. Taking together, it suggested that PASET could directly whittle down obese persons’ abdominal fat mass.

**Table 6:** The thickness of abdominal subcutaneous fat tissue using ultrasound measurement

	Upper abdomen (mm)		Lower abdomen (mm)	
	Before treatment	After treatment	Before treatment	After treatment
PASET group	40.95 ± 7.03	37.63 ± 5.82**	38.86 ± 6.96	35.37 ± 7.11**
Control group	35.29 ± 5.42	36.94 ± 5.54	35.93 ± 6.24	36.25 ± 8.46

Statistical significant difference between before and after treatment in each group (** p < 0.01)

### PASET is a relative safe method for obesity treatment

After we completed the clinical trial, we systemically evaluate the safety of PASET. The results indicated that subcutaneous congestion on abdomen of two participants happened, and it disappeared after 1 week. Erythematous subcutaneous nodule taken place in one person’s abdomen and it regressed spontaneously after 2 weeks, and left slight postinflammatory hyperpigmentation 2 month later, which eventually disappeared 1 month later. By the evaluation of VAS questionnaire on pain feeling, no patients withdrew from the trial due to insufferable pain, when they accepted the performance of PASET. The results suggest that PASET is a relative safe therapeutic technology for simple obesity.

## Discussion

In present study, we use polyglycolic acid sutures instead of traditional catgut to embed in abdominal acupoints for simple obesity treatment. It shows that the novel therapeutic technology significantly reduces body weight, BMI, HC, WC, WHR, WtHR and the thickness of abdominal subcutaneous fat tissue, improves evaluated scores of physical function, self-esteem, public distress and sexual life, decreases blood pressure, glycemia, LDL, uric acid and increases HDL, as well as down-regulates the levels of TNF-α and IL-1β in obese persons. The efficacies of PASET are superior to lifestyle modification.

The increasing prevalence of overweight and obese adults has become a public concern problem. Obesity not only causes, or aggravates some pathological progress, such as cerebral-cardiovascular disease, diabetes, gallstones and musculoskeletal diseases, but also influence quality of life including depression, anxiety, stress, lower self-esteem and sexual dysfunction [25]. IWQOL-Lite is a self-evaluated report consisting of a total score and scores on each of five scales, which exhibits strong psychometric properties and clinically sensitive brief measure of quality of life in obese persons [20, 26]. In current study, 51 participators completed the IWQOL-Lite, and PASET group exerts main therapeutic effects on amelioration of quality of life by enhancing total scores and scores of four areas in physical function, self-esteem, sexual life and public distress after treatment. However, control group fails to suggest advantageous efficacy on improvement of quality of life using lifestyle modification at the end of the treatment. Due to obese persons living in their houses, it is difficult to accurately assess the food intake although hypocaloric diets menu had been established, as well as if the intensity and time of exercise were accordance with the physical requirement. The limitations should be avoided by effective monitoring methods followed by trial.

Long-term abdominal obesity causes alterations of hormonal, inflammatory and endothelial level, which contribute to the hypertensive state [27]. Excessive adipose tissue release free fatty acid (FFA) resulted in obesity-related dyslipidemia. The enhanced FFA delivers into live and produces hypertriglyceridemia as well as low concentration of HDL and high concentration of LDL [28]. LDL cholesterol has been crucial actual atherogenic risk, since a high concentration of small, dense LDL cholesterol closely associates with a high prevalence of cardiovascular disease [29]. Our study indicates that PASET either lows values of diastolic and systolic pressures in obese persons, or enhances HDL concentration and decreases LDL level. Although both of PASET and control groups show slight decline of triglyceride concentration, there is no statistical significance after treatment. Nevertheless, the beneficial efficacy of PASET on dyslipidemia and blood pressure contributes to reduction of cerebral-cardiovascular disease risk factors. In addition, we also find PASET notably reduces uric acid level. Whether the elevated serum uric acid is casual or a consequence of obesity confuse researchers for a long time. Zheng and colleagues demonstrated that high serum uric acid levels increased the risk of obesity by a longitudinal 4411 obese subjects clinical trial [30]. Therefore, PASET decreases the level of serum uric acid is benefit to further attenuation of obese risk.

Evidence suggests that chronic low-grade inflammation in adipose tissue plays a predominant role in the development of obesity-related metabolic dysfunction [31]. In the obese state, necrotic adipocyte recruits macrophages to secret pro-inflammatory cytokines, such as TNF-α, IL-1β, IL-6, MCP-1, which potentially arouses obesity-related insulin resistance [28]. In order to interrupt the pathological progress of T2DM onset in obese persons, anti-inflammatory response is a necessary therapeutic approach [32]. Our study showed that PASET distinctly reduced the concentrations of TNF-α and IL-1β, two powerful pro-inflammatory cytokines, as well as decreased IL-6, MCP-1 levels although there was no statistical difference between them. It suggest PASET may regulate macrophage polarization from pro-inflammatory phenotypic to anti-inflammatory status. Interestingly, we found IL-18 level slightly ascends after PASET. Some studies evidence that circulating increased IL-18 associates with obese subjects and metabolic syndrome [33, 34]. However, knockout mice deficient in IL-18 or IL-18 receptor cause hyperphagia, obesity and insulin resistance, and it is worth reassessing the role of IL-18 in the pathogenesis of obesity and insulin resistance [28, 35]. We hypothesize the change of IL-18 level in our study may contribute to the energy expenditure via PASET for obese persons, because Ye and McGuinness think inflammation during obesity is not all bad and promoted IL-18 may exerts a protective effect on limiting adiposity [36]. Of course, precision mechanism needs to be investigated in next experiment.

In the evaluation of safety on simple obesity using PASET, our results indicate the therapeutic technology is relative safe. Only one participator subjected to erythematous subcutaneous nodule on embedding site of abdomen, and the nodule and hyperpigmentation eventually disappeared after 3.5 months. Hsu and colleagues reported that erythematous swelling and nodules appeared in the acupuncture points on abdomen of a 27-year-old woman who received the acupuncture point catgut embedding treatment, and the complications disappeared 6 months later [37]. It suggests the complications of PASET can quickly attenuate compared with those of catgut embedding treatment. In addition, participators also can tolerate the pain feeling during the process of PASET and no one withdraws the trial due to pain or discomfort.

## Conclusions

Based on the advantageous actions on ameliorations of quality of life, metabolic profile and inflammatory response by PASET, it contributes to weight loss, which embodies the decreases of weight, BMI, HC, WC, WHR, WtHR as well as abdominal subcutaneous fat thickness by scanning of B-model ultrasound. Therefore, we think PASET is an effective therapeutic approach in the treatment of simple obesity.

## Data Availability

The datasets used during the current study are available from the corresponding author on reasonable request.

## Abbreviations

ACE: acupoint catgut embedding
BMI: body mass index
ELISA: enzyme-linked immunosorbent assay
FFA: free fatty acid
HC: hip circumference
HDL-C: high density lipoprotein cholesterol
IWQOL-Lite: Impact of Weight on Quality of Life Questionnaire
IL-6: interleukin-6
IL-18: interleukin-18
IL-1β: interleukin-1beta
LDL-C: low density lipoprotein cholesterol
MCP-1: monocyte chemoattractant protein-1
PASET: polyglycolic acid sutures embedding therapy
TC: total cholesterol
T2DM: type 2 diabetes mellitus
TG: triglyceride
THR: target heart rate
TNF-α: tumor necrosis factor-alpha
WC: waist circumference
WHO: World Health Organization
WHR: waist/hip ratio
WtHR: waist-to-height ratio
